# Differentiation Syndrome in Acute Myeloid Leukemia: Molecular Mechanisms, Clinical Spectrum, and Emerging Therapeutic Paradigms

**DOI:** 10.3390/ijms27041775

**Published:** 2026-02-12

**Authors:** Razan Mansour, Abeer Yaseen, Zaid Abdel Rahman

**Affiliations:** 1 Department of Hematology and Medical Oncology, Yale School of Medicine, New Haven, CT 06510, USA; 2 Department of Internal Medicine, King Hussein Cancer Center, Amman 11941, Jordan; abeeryassin2@gmail.com; 3 School of Medicine, University of Jordan, Amman 11942, Jordan

**Keywords:** acute myeloid leukemia, differentiation, differentiation syndrome, IDH1/2 inhibitors, FLT3 inhibitors, menin inhibitors

## Abstract

Acute myeloid leukemia (AML) is characterized by differentiation arrest, driving blast proliferation, and abnormal blood formation. While differentiation therapy revolutionized acute promyelocytic leukemia (APL) with all-trans retinoic acid (ATRA) and arsenic trioxide (ATO), its extension into non-APL AML has been limited until recent targeted agents. This narrative review synthesizes preclinical and clinical evidence into differentiation-inducing therapy, with a focus on IDH1/2, FLT3 and menin inhibitors. Following SANRA guidelines, we searched PubMed (2010–September 2025) for clinical trials and key preclinical studies, with particular attention to the molecular mechanism of differentiation induction, clinical efficacy, and the management of differentiation syndrome (DS). IDH1/2 inhibitors (ivosidenib, enasidenib, olutasidenib) yield overall response rates (ORRs) of 30–94% in AML with DS in 10–19%. Menin inhibitors (revumenib, ziftomenib, enzomenib, bleximenib) achieve ORRs of 33–88% in KMT2A-rearranged or *NPM1*-mutated AML, with DS in 10–25% and QT prolongation as key toxicities. *FLT3* inhibitors (gilteritinib, quizartinib) improve survival in *FLT3*-mutated AML with DS in 1–5%. Resistance mutations limit durability and combinations enhance efficacy. Differentiation therapy represents a paradigm shift towards non-cytotoxic AML management. Improved recognition of DS and rational combination approaches will be essential to maximize the therapeutic benefit. Future research should address mechanisms of resistance and biomarkers to achieve cures beyond APL.

## 1. Introduction

Acute myeloid leukemia (AML) is a clonal malignancy of hematopoietic stem and progenitor cells characterized by the uncontrolled expansion of immature blasts and failure of normal hematopoiesis [1]. At a biological level, AML is driven by the disruption of the genetic and epigenetic regulation that guide myeloid differentiation [2], resulting in persistent self-renewal and arrest of leukemic cells at an immature developmental stage [3]. The paradigm of “differentiation therapy” was first established in acute promyelocytic leukemia (APL), a distinct AML subtype driven by the PML–RARA fusion oncoprotein which enforces the transcriptional repression of myeloid differentiation [4].

The introduction of all-trans retinoic acid (ATRA) in the mid-1970s demonstrated that the pharmacological release of this block can induce terminal differentiation of leukemic promyelocytes into mature granulocytes, leading to high remission rates [4]. Subsequent combination of ATRA and arsenic trioxide (ATO) achieved durable remission and long-term cure rates exceeding 90–95% in APL, despite minimal reliance on conventional cytotoxic chemotherapy 5. This paradigm-shifting success established differentiation therapy and raised the question of whether similar approaches could be extended into non-APL AML [5,6,7,8].

Early attempts to generalize differentiation therapy using ATRA were largely unsuccessful. Meta-analysis of five randomized controlled trials including more than 1000 patients with non-APL AML demonstrated no significant improvement in overall survival (OS) or remission rates [9]. While preclinical studies suggested that certain molecular subsets might respond to ATRA [10,11], these findings have not translated into clear clinical benefits in non-APL AML, underscoring the molecular specificity underlying APL’s unique sensitivity and highlighting the need for mechanism-driven approaches.

Historically, treatment for non-APL AML was predominantly based on intensive cytotoxic chemotherapy, primarily consisting of a backbone of cytarabine- and anthracycline-based regimens established in the 1970s, designed to eliminate proliferating leukemic blasts, without addressing the underlying differentiation blockade that drives disease pathogenesis. While these strategies achieved complete remission (CR) rates of 60–80% in younger adults and 40–60% in older patients [12,13], long-term outcomes remained poor, particularly in older adults and those with adverse-risk disease, with five-year overall survival rates typically below 30%. The advent of next-generation sequencing (NGS) and large-scale genomic profiling has since revealed recurrent genetic and epigenetic mutations that directly contribute to differentiation arrest, enabling the development of targeted agents designed to reverse these effects.

Differentiation has therefore re-emerged as a central focus of targeted therapy development in non-APL AML [14]. Preclinical evidence demonstrated that leukemic cells could be induced to undergo maturation through the targeted modulation of oncogenic transcriptional networks and epigenetic regulators [15]. More recently, novel menin–*KMT2A* inhibitors have shown efficacy in mouse xenograft models harboring *KMT2A*-rearranged or *NPM1*-mutated AML, and they have shown robust differentiation-inducing activity [16], validating differentiation therapy as a viable strategy beyond APL.

### 1.1. Molecular Mechanisms of Differentiation Induction by Targeted Agents in Non-APL AML

In non-APL AML, differentiation arrest stems from oncogenic disruptions of epigenetic and signaling pathways that block normal myeloid maturation. Mutant *IDH1/*2 enzymes gain neomorphic activity, converting α-ketoglutarate to the oncometabolite (R)-2-hydroxyglutarate (2-HG), which inhibits α-ketoglutarate-dependent dioxygenases, causing hypermethylation and maturation blockade [17,18]. Selective inhibition with enasidenib suppresses 2-HG, induces differentiation in primary *IDH2*-mutated AML cells ex vivo and xenografts, and improves survival in aggressive models. Clinical correlates show that 2-HG reduction precedes response, with the normalization of progenitors, emergence of functional mature myeloid cells, and differentiation even with persistent mutant allele burden, confirming differentiation as the primary efficacy mechanism [19,20].

In menin-dependent AML (*KMT2A*-rearranged, *NPM1*-mutated, *NUP98*-fused, and related subsets), menin inhibitors disrupt the menin–*KMT2A* interaction, leading to the loss of menin and *KMT2A* from chromatin at key loci, downregulation of oncogenic HOXA/MEIS1 genes, upregulation of differentiation genes, proliferation arrest, and apoptosis, yielding a differentiated phenotype in sensitive leukemias [21].

For *FLT3*-mutated AML, gilteritinib relieves constitutive signaling, promoting terminal myeloid differentiation in relapsed/refractory (R/R) cases. In clinicopathologic analysis, responders showed differentiation, with stable/hypercellular marrow, left-shifted granulocytic hyperplasia, reduced blasts, emergence of mature granulocytic/monocytic populations on flow cytometry, and often persistent *FLT3* mutant allele, distinguishing this from cytotoxic clearance [22].

### 1.2. Differentiation Syndrome, Definition, Diagnosis, and Management

Differentiation syndrome (DS) is an on-target inflammatory complication resulting from the rapid maturation of leukemic blasts, cytokine release, endothelial activation, and capillary leak following exposure to differentiation-inducing therapies. While classically described in APL, DS is increasingly recognized in non-APL AML treated with *IDH*1/2 inhibitors, menin inhibitors, and, less commonly, *FLT3* inhibitors. Clinically, DS typically presents with otherwise unexplained fever, dyspnea or hypoxia, rapid weight gain (>5 kg), peripheral edema, pleural or pericardial effusions, hypotension, acute renal dysfunction, and pulmonary infiltrates on imaging. Leukocytosis or hyperleukocytosis may accompany DS but is not required for diagnosis. The timing of onset varies by therapeutic class, occurring at a median of 17–48 days with *IDH* inhibitors, within 1–4 weeks with menin inhibitors, and ranging from days to months with *FLT3* inhibitors.

Diagnosis remains clinical and is generally based on criteria adapted from APL-associated DS, most commonly the Montesinos criteria, which require the presence of at least two compatible clinical features in the absence of an alternative explanation such as infection, heart failure, or disease progression [20].

The management of DS emphasizes early recognition and prompt intervention. Unlike in high-risk APL, where prophylactic corticosteroids are routinely used to mitigate risk, routine prophylaxis is not recommended for non-APL AML treated with *IDH1/2*, menin, or *FLT3* inhibitors due to the often delayed and unpredictable onset of DS in these settings. Systemic corticosteroids, most commonly dexamethasone 10 mg intravenously twice daily, should be initiated immediately upon clinical suspicion and tapered over days to weeks once symptoms resolve. Supportive measures include diuretics for fluid overload, hydroxyurea for marked leukocytosis, oxygen or ventilatory support as needed, and temporary interruption of the differentiating agent in moderate to severe cases, although permanent discontinuation is rarely required. These management strategies, adapted from established APL guidelines, have proven effective and safe in non-APL AML treated with *IDH1/2*, menin, and *FLT3* inhibitors, with low DS-related mortality when addressed expeditiously [23].

In this article, we provide an overview of the current mechanisms of differentiation arrest in AML and the ways in which emerging targeted treatments exploit this hallmark. We focus on the clinical development of differentiation-directed therapies, notably *IDH*1/2 inhibitors, *FLT3* inhibitors, and menin inhibitors, as these agents have demonstrated the ability to induce blast differentiation in clinical trials. We focus on the clinical development, clinical efficacy, key clinical trials, differentiation-associated toxicities, and the resistance pathways that limit their long-term success. Finally, we discuss future directions aimed at enhancing differentiation-based therapy through rational combination regimens and next-generation inhibitors to overcome resistance and improve durable remission rates.

## 2. Results

### 2.1. Targeting IDH1/2

*IDH*1/2 inhibitors are small molecules that induce differentiation by binding to the active site of the mutant isocitrate dehydrogenase enzyme. The bond also blocks the production of 2-hydroxyglutarate and restores normal levels of α-Ketoglutarate (α-KG), thereby reactivating epigenetic regulation and removing the differentiation block. Across clinical trials, DS has been reported in 12–19% of patients receiving *IDH*1/2 inhibitors [24], with a median time to onset of DS of 17–20 days. Most cases of DS related to *IDH* inhibitors have a mild to moderate severity and are usually manageable with steroids, supportive care, and temporary treatment interruption. Table 1 shows a summary of *IDH*1/2 inhibitors and the clinical efficacy incidence of major treatment-related adverse events with a focus on differentiation syndrome.

Ivosidenib (Servier Pharmaceuticals LLC, Suresnes, France), a selective *IDH*1 inhibitor targeting the R132 mutation, demonstrated meaningful activity in R/R AML. In the initial phase 1 dose-escalation and dose-expansion study of ivosidenib monotherapy in *IDH*1-mutated AML [25], the overall response rate (ORR), complete remission or complete remission with incomplete hematologic recovery (CR/CRi), and CR rates were 41.6%, 30.4%, and 21.6%, respectively. The duration of response was highest in patients who achieved CR, with a median of 9.3 months. Among patients who achieved CR/CRi, 21% had no residual *IDH*1 mutation detected after therapy. Treatment-related adverse events (TRAEs) of grade 3 or higher that were defined to be of special interest were prolongation of the QT interval (7.8%, any grade 24%), DS (3.9%, any grade 10.6%), and leukocytosis (1.7%, any grade 36%). Median time to onset of DS was 29 (range 5 to 59) days. None were grade 4, and no patients discontinued the medication due to DS. Treatment for DS included glucocorticoids, diuretics, and (if accompanied by leukocytosis) hydroxyurea. With these interventions, the syndrome was resolved in 17 of 19 patients, and the remaining 2 patients had ongoing DS at the data-cutoff date. However, since this was a first-in-human experience where the signs and symptoms were not initially recognized as DS, and due to a lack of a codified adverse event terms for DS outside of the context of APL or ATRA, the FDA suspected that DS was underreported in this setting, and subsequently sought to perform a systematic analysis of DS cases based on adverse event terms, laboratory abnormalities, and vital sign results grouped per Montesinos criteria [26]. The algorithm identified potential DS in 40% (72/179) of patients treated with ivosidenib; however, the subsequent review by the FDA showed that around half of these cases were DS secondary to ivosidenib (34/179, 19%) [24].

In the phase III AGILE trial [27], patients with newly diagnosed *IDH*1-mutated AML who were ineligible for intensive chemotherapy were randomly assigned to receive oral ivosidenib and subcutaneous or intravenous azacitidine or to receive a matched placebo and azacitidine. The primary endpoint was event-free survival (EFS), defined as treatment failure, relapse from remission, or death. In the intention-to-treat population (N = 146), EFS was significantly higher in the ivosidenib-and-azacitidine group (HR 0.33; 95% confidence interval [CI], 0.16 to 0.69; *p* = 0.002). The median overall survival (OS) was 24 months (95% CI, 11.3 to 34.1) and 7.9 months (95% CI, 4.1 to 11.3) in the ivosidenib-and-azacitidine and placebo-and-azacitidine groups, respectively (HR = 0.44; 95% CI, 0.27 to 0.73; *p* = 0.001). As for safety, the percentage of patients with DS of any grade was 14% with ivosidenib and azacitidine (no grade ≥ 4 events) and, interestingly, 8% with placebo and azacitidine (including one grade 4 event), with a median time to onset of 19.5 (3–33) days. No deaths due to DS were noted in either group.

Ivosidenib has also been evaluated as part of a triplet regimen in combination with azacitidine and venetoclax in patients with newly diagnosed (ND) *IDH*-mutated AML, not fit for intensive chemotherapy, or high-risk myelodysplastic syndrome (MDS) or myeloproliferative neoplasm (MPN) (defined as ≥10% blasts or intermediate/high risk by IPSS, R-IPSS, or D-IPSS). The ORR was 94% with 93% achieving CRc within five cycles and 77% achieving minimal residual disease (MRD) negativity by flow cytometry. These responses translated into a 3-year OS for MDS or MPN (n = 12), newly diagnosed AML (n = 31), and R/R AML (n = 13) subsets of 81.5% (95% CI: 61.1–100%), 71.4% (53.2–95.8%), and 52.1% (28.8–94.3%), respectively. Patients who received an allogeneic stem cell transplant (allo-SCT) had a 3-year OS of 94.7% (95% CI: 85.2–100%) compared to 52.8% (25.6–78.2%) in those who did not [28,29].

Enasidenib (Bristol Myers Squibb, Princeton, NJ, USA), an oral, selective inhibitor of mutant-*IDH*2 enzymes, has demonstrated efficacy in the management of R/R and ND *IDH*2-mutated AML. In a phase I/II study, enasidenib doses of 50 to 650 mg per day were evaluated, and a once-daily 100 mg dose was selected on the basis of the pharmacokinetic and pharmacodynamic profiles, as the maximum tolerated dose was not reached. DS occurred in 23 (8%) patients with 15 having grade 3–4 DS. Median time to onset was 48 days (range 10–340 days). Enasidenib dosing was interrupted in 10 patients with DS, but permanent drug discontinuation was not required. Enasidenib was also associated with non-infectious leukocytosis in 17%, primarily within the first two cycles; however, these were not necessarily accompanied by DS. In regard to response, the ORR for all R/R AML was 40.3% with 19.3% of patients attaining CR. The median time to first response was 1.9 months (range, 0.5–9.4 months) with 87.3% of responding patients attaining a first response by cycle 5 [30]. In patients with newly diagnosed AML, the ORR was 30.8% with 21% achieving CR/CRi [31].

In a randomized phase II trial, patients received enasidenib plus azacitidine or azacitidine only; in the enasidenib plus azacitidine arm, 7% achieved a response, compared with 36% in the azacitidine monotherapy group. Of note, the rate of DS in the enasidenib plus azacitidine arm was 10% [32].

Olutasidenib (Rigel Pharmaceuticals, Inc., San Francisco, CA, USA) is a structurally distinct allosteric non-competitive *IDH*1 inhibitor that is FDA-approved for the treatment of adult patients with R/R *IDH*1-mutated AML [33]. Olutasidenib has demonstrated clinically meaningful efficacy in a pivotal phase 2 trial involving 147 efficacy-evaluable patients with R/R *IDH*1-mutated AML [34,35]. The ORR was 48%, while the CR/CRi rate was 35% with a median duration of response of approximately 25.9 months. Median OS was 11.6 months, which included patients who had failed prior venetoclax-based regimens [36]. DS occurred in approximately 14% of patients, with grade ≥ 3 events in 9% and one fatal case reported. Olutasidenib has also been studied in combination with azacitidine in *IDH*1-mutated MDS, yielding an ORR of 59% and a CR/CRi rate of 27% with durable remissions and a tolerable safety profile, DS occurred in three patients (14%), including one (5%) with grade 3 severity [37,38].

### 2.2. Targeting Menin

The menin and histone-lysine-N-methyltransferase 2A (*KMT2A*) protein complex is an essential epigenetic regulator of genes (e.g., MEIS1 and the homeobox [Hox] gene family) essential for leukemic self-renewal [39]. This is particularly pronounced in *NPM1*-mutated AML (approximately 25–30% of AML) as well as *KMT2A*-rearranged AML (5–10% of AMLs) [40]. In these genetically defined subtypes, menin facilitates the oncogenic transcriptional program leading to myeloid differentiation in preclinical models. Table 2 shows a summary of clinical studies evaluating menin inhibitors and the clinical efficacy incidence of major treatment-related adverse events with a focus on differentiation syndrome.

### 2.3. Revumenib

Revumenib is a first in class oral menin inhibitor. It demonstrated robust differentiation-driven activity in the phase I/II AUGMENT-101 trial involving R/R *KMT2A*-rearranged or *NPM1*-mutated AML. Among the 161 patients enrolled, the ORR reached 64% in the *KMT2A*-rearranged cohort and 47% in the *NPM1*-mutated group. Rates of CR/CRi were identical between cohorts at 23%. MRD-negativity among responders was achieved in 58% of *KMT2A*-rearranged patients and 64% of those with *NPM1* mutations. A total of 36% of the responding *KMT2A*-rearranged patients and 17% of *NPM1*-mutant responders received allo-SCT [41,42].

Revumenib demonstrated a manageable and distinct safety profile; the most frequently reported TRAEs of any grade included nausea (28%), vomiting (18%), increased alanine aminotransferase (ALT) levels (18%), anemia (16%), febrile neutropenia (14%), and QT interval prolongation (16%). Among grade 3 or higher TRAEs, febrile neutropenia occurred in 14% of patients, and QTc prolongation ≥ grade 3 was observed in 16% of patients, including one case of dose-limiting QT prolongation beyond 500 ms. QTc prolongation typically peaked around day 8 of treatment but was not associated with any documented arrhythmia or treatment-related deaths, and it was managed with monitoring and dose adjustments. DS was reported in 16% of patients, all of which were grade 2 or 3 in severity. These cases responded to corticosteroids and/or hydroxyurea without the need for permanent treatment discontinuation or dose reduction, and no DS-related mortality occurred. Overall, 6% of patients discontinued therapy due to adverse events.

Revumenib has also been evaluated in combination with azacitidine and venetoclax in a phase I dose-escalation and -expansion study at two dose levels (113 mg or 163 mg orally every 12 h in combination with strong cytochrome P450 inhibitor azoles) in patients aged 60 years and older newly diagnosed with AML with *NPM1* mutation or *KMT2A* rearrangement; 43 patients were enrolled and treated with a CR/CRi rate of 81.4% (*NPM1*-mutated: 79.4%; *KMT2A*-rearranged: 88.9%) with 84% of evaluable patients achieving remission within one cycle of therapy. There were no dose-limiting toxicities. DS occurred in eight (19%) patients and QTc prolongation in nineteen (44%) patients; however, neither required permanent discontinuation of revumenib.

Similarly, revumenib was evaluated in an all-oral combination with decitabine/cedazuridine and venetoclax in ND patients with *NPM1*-mutated, *KMT2A*-rearranged, or NUP98-rearranged AML or mixed-lineage acute leukemia who were not candidates for high intensity chemotherapy. Among evaluable pts, the CR rate was 88% (14/16 pts, 95% CI, 59–94), with MRD-negative rate by flow cytometry of 100%. At a median follow-up of 6 months (range, 1–14), the median OS and EFS were not reached. Allo-SCT has been performed in five (29%) pts: two (18%) *NPM1*-mutated and three (50%) *KMT2A*-rearranged AML. Relapse occurred in two pts (1 *NPM1*-mutated and 1 *KMT2A*-rearranged). Neither underwent allo-SCT in CR1 and both had detectable MEN1 M327I mutations at relapse. The most common TRAEs were infection in nine patients (53%), all grade 3. QTc prolongation occurred in eight (47%) patients; three were grade 2 (18%) and the rest were grade 1 (29%). DS occurred in four (24%) patients; two patients had grade 3 DS which promptly resolved with steroids [43].

### 2.4. Ziftomenib

Ziftomenib, another oral menin inhibitor, has shown activity across many preclinical and clinical settings. In the phase 1/2 open-label multicenter KOMET-001 trial, 92 patients with R/R *NPM1*-mutated AML were treated with ziftomenib 600 mg once daily, with a CR/CRi rate of 22% and a median duration of response of 4.6 months. In regard to TRAEs, 86 of 92 patients (93%) had grade ≥ 3 treatment-emergent adverse events, with the most common of these being febrile neutropenia (26%), anemia (20%), thrombocytopenia (20%), and QT prolongation (9%). DS occurred in 25% of patients, but grade ≥ 3 DS occurred in only 15% (all grade 3, no grade 4 or 5). Two patients (2%) discontinued treatment because of ziftomenib-related DS; one patient had resolved DS but stopped therapy because of other unrelated complications; the other died due to progressive AML.

Ziftomenib is also under evaluation in the ongoing KOMET-007 phase1a/1b, combining it with venetoclax/azacitidine, venetoclax alone, or cytarabine and daunorubicin (7+3) in *NPM1*-mutated or *KMT2A*-rearranged AML. In an interim analysis for the intensive chemotherapy arm (n = 46 evaluable) [44], CR rates were 88% (30/34) for *NPM1*-mutated and 83% (10/12) for *KMT2A*-rearranged; composite complete remission (CRc) rates were 94% (32/34) and 83% (10/12), respectively. The most common grade ≥ 3 TEAEs were febrile neutropenia (47%), thrombocytopenia (31%), anemia (22%), and neutropenia (20%). Importantly, there were no cases of DS, ziftomenib-associated QTc prolongation, or drug-limiting toxicities (DLTs) with the 200 mg or 400 mg dose levels.

Ziftomenib was also evaluated in combination with azacitidine and venetoclax in ND and in R/R *NPM1*-mutated or *KMT2A*-rearranged AML. In the ND *NPM1*-mutated cohort, the recommended phase 2 dose (RP2D) of ziftomenib 600 was used (n = 31, evaluable); the CRc rate was 84% (26/31) after a median time to first CRc of 3.5 weeks (range 2.4–9.4), with local MRD-negativity rates among the tested CRc responders of 54% (13/24) after a median time to first MRD-negativity of 8.4 weeks (range 2.9–17.4). DS occurred in one (3%) patient (grade 2), which successfully resolved with protocol-specified mitigation. One patient (3%) had investigator-assessed ziftomenib-associated QTc prolongation (grade 3); however, there were concomitant significant electrolyte abnormalities, and the event resolved with electrolyte repletion [45].

In the R/R AML cohort (n = 70 evaluable patients, *NPM1*-mutated = 43; *KMT2A*-rearranged = 27), with a median follow-up of 18.0 weeks for *NPM1*-mutated and 16.4 weeks for *KMT2A*-rearranged, the ORRs were 65% (28/43) for *NPM1*-mutated and 33% (9/27) for *KMT2A*-rearranged; CRc rates were 49% (21/43) for *NPM1*-mutated and 22% (6/27) for *KMT2A*-rearranged after median time to first CRc of 4.9 weeks (range 2.7–15.6) and 5.5 wks (range 2.6–18.9), respectively; MRD-negativity rates among the tested CRc responders were 50% (9/18) for *NPM1*-mutated and 60% (3/5) for *KMT2A*-rearranged after median time to first MRD-negativity of 5.9 weeks (range 2.9–15.6) and 8.1 weeks (range 7.7–18.9), respectively. DS occurred in one (1%) *NPM1*-mutated patient (grade 3), which lasted 1 day and successfully resolved with protocol-specified DS mitigation. No ziftomenib-related QTc prolongation was reported with the combination, and no DLTs were observed in phase 1a [46].

**Table 2:** Menin inhibitors.

Medication	Trial Name/ID	Line of Therapy	Response Rate (ORR/CR/CRh)	Grade ≥ 3 Complications	Differentiation Syndrome (DS)
Revumenib	AUGMENT-101	R/R AML (*KMT2A*r/NPM1m)	*KMT2A*r: ORR 64% *NPM1*m: ORR 47%	QTc Prolongation: 16% Febrile Neutropenia: 14%	16% (Any grade) All G2–3
Revumenib + Aza + Ven	Beat AML (NCT03013998)	ND AML (Older adults)	CR/CRh 81.4% ORR: 88.4% Universal MRDnegativity in responders	QTc Prolongation: 12% (G3)	19% (Any grade) 5% (Grade 3)
Revumenib + Dec/Ced + Ven	SAVE Study	ND AML (*NPM1*m/*KMT2A*r)	CR: 88% MRD-negative Rate: 100%	Infection: 53% Febrile Neutropenia: 37%	24% (Any grade) 12% Grade 3
Ziftomenib	KOMET-001	R/R *NPM1*m AML	CR/CRh: 22% mDOR: 4.6 months	Febrile Neutropenia: 26% Anemia/Thrombocytopenia: 20%	25% (Any grade) 15% Grade 3
Ziftomenib + 7+3	KOMET-007 [39]	ND AML (*KMT2A*r/*NPM1*m)	*NPM1*m: CR 100% *KMT2A*r: CR 83%	Febrile Neutropenia: 15% Thrombocytopenia: 15%	2% (Any grade) 1 Case G3 reported
Ziftomenib + Aza + Ven	KOMET-007 [40]	ND *NPM1*m AML	CRc: 84% MRD-negativity Rate: 54%	QTc Prolongation: 3% (G3)	3% (Grade 2)
Ziftomenib + Aza + Ven	KOMET-007 [41]	R/R AML (*KMT2A*r/*NPM1*m)	*NPM1*m: ORR 65% *KMT2A*r: ORR 33%	Thrombocytopenia: 31% Anemia: 26%	1% (Grade 3)
Enzomenib	Phase 1/2 [47]	R/R Acute Leukemia	*KMT2A*r: ORR 72.7% *NPM1*m: ORR 47%	No ≥G3 QTc prolongation Sepsis: 25%	12.9% (Any grade) 7.7% Grade 3/4
Enzomenib + Aza + Ven	Phase 1 [48]	R/R AML	CRc: 56% MRD-negativity Rate: 83%	Thrombocytopenia: 44.4% Leukopenia: 38.9%	1 Case (Grade 2)
Bleximenib + Ven (+/− Aza)	ALE1002	R/R AML (*KMT2A*r/*NPM1*m)	ORR: 69–79% CRc: 38.5%	Febrile Neutropenia: 37% Anemia: 46.7%	6% (Any grade) G5 reported at 50 mg
Bleximenib + 7+3 Chemo	ALE1002	ND AML (*KMT2A*r/*NPM1*m)	ORR: 95.8% CR: 87.5%	Thrombocytopenia: 79.5% Neutropenia: 72.7%	Low (Safety mitigation in place)

Data compiled from AUGMENT-101 (Revumenib), Beat AML/SAVE (Revumenib combos), KOMET-001/007 (Ziftomenib), and ALE1002 (Bleximenib). Abbreviations: 7+3: Cytarabine and Anthracycline induction; AML: Acute Myeloid Leukemia; Aza: Azacitidine; CR: Complete Remission; CRc: Composite Complete Remission (CR + CRh + CRp + CRi); CRh: Complete Remission with partial hematologic recovery; Dec/Ced: Decitabine/Cedazuridine; DS: Differentiation Syndrome; *KMT2A*r: *KMT2A* rearrangement; mDOR: Median Duration of Response; MRD: Measurable Residual Disease; ND: Newly Diagnosed; *NPM1*m: *NPM1* mutation; ORR: Overall Response Rate; QTc: Corrected QT interval; R/R: Relapsed/Refractory; Ven: Venetoclax. Safety: Adverse events graded by CTCAE. Differentiation syndrome (DS) rates reflect investigator-reported or trial-adjudicated events. G5 refers to fatal adverse events.

### 2.5. Enzomenib

Enzomenib (DSP-5336) is an oral menin–*KMT2A* inhibitor, intentionally designed with different physicochemical properties, such as a short half-life of 2–5 h, low lipophilicity, and rapid drug clearance. In a phase1/2 trial in patients with R/R *KMT2A*-rearranged, *NPM1*-mutated, and other HOXA9/MEIS1-driven leukemias [47], 116 heavily pre-treated patients were enrolled; they had a median of two prior treatment regimens, with a range (1–9); 36 (31%) patients had prior allo-SCT and 86 (74.1%) patients had prior venetoclax. *KMT2A* rearrangement was documented in 61 pts (52.6%), *NPM1* mutation in 34 (29.3%), and other abnormalities in 21 (17.7%). TRAEs included nausea (16.4%), DS (12.9%, grade 3 DS was reported in eight pts (6.9%), grade 4 in one patient (0.8%)), and vomiting (11.2%). There was no G3+ treatment-related QT prolongation. Grade 1/2 treatment-related QT prolongation was reported in five pts (4.3%), which did not require discontinuation, and was complicated by underlying electrolyte abnormalities and concomitant medications. Outcomes were reported for patients without prior exposure to menin inhibitors. The ORR and CR + CRi rates at the recommended phase 2 dose (RP2D) for pts with *KMT2A*-rearranged (300 mg BID with strong CYP3A4 inhibitor azoles) were 72.7% (8/11) and 45.5% (5/11). Dose optimization for the *NPM1* cohort is still ongoing.

Enzomenib was also evaluated in a phase 1 study in combination with venetoclax and azacitidine in patients with R/R AML [48]. Among the 18 enrolled patients (median age 50 (range: 21–76), median two prior regimens, 16.7% prior allo-SCT, 33.3% prior venetoclax exposure, 27.8% prior menin inhibitor exposure), *KMT2A* rearrangement and *NPM1* mutation were documented in 7 (38.9%) and 11 (61.1%) pts, respectively. The CRc rate was 56%; of the 15 responders, 12 were assessed for MRD and 83% (10/12) were MRD-negative by flow cytometry or NGS. Of the patients with prior exposure to venetoclax, 67% (6/9) achieved a CRc.

### 2.6. Bleximenib

Bleximenib (JNJ-75276617) is another oral menin inhibitor which was evaluated in the phase 1 ALE1002 study, where it showed high rates of response at the bleximenib RP2D 100 mg BID in combination with venetoclax and azacitidine in patients with ND *KMT2A*-rearranged or *NPM1*-mutated AML. Bleximenib is currently being evaluated in cAMeLot-2 (NCT06852222), a phase 3, randomized, double-blind, placebo-controlled, global multicenter study that will evaluate the efficacy and safety of bleximenib with venetoclax + azacitidine in adults with ND *KMT2A*-rearranged or *NPM1*-mutated AML who are ineligible for intensive chemotherapy, and the HOVON 181 AML/AMLSG 37-25, a double-blinded, phase 3 study of bleximenib or placebo in combination with standard induction and consolidation therapy followed by maintenance for the treatment of patients with ND *KMT2A*-rearranged or *NPM1*-mutated AML eligible for intensive chemotherapy.

### 2.7. Targeting FLT3

The incidence of DS with *FLT3* inhibitors is less frequent than in *IDH* and menin inhibitors and is estimated to be in the range of 1–5% across various studies [49]. Patients who develop *FLT3*-associated DS tend to present with prominent dermatologic manifestations (rash, neutrophilic dermatoses), yet similar to IDH-DS, they tend to occur within days but can also be delayed, presenting weeks to months later. There have been no reports in the literature of DS occurring with midostaurin, likely related to the use of midostaurin in combination with intensive chemotherapy as outlined in the RATIFY trial, thereby mitigating the risk of DS [50].

### 2.8. Gilteritinib

Gilteritinib is a highly selective, oral *FLT3* inhibitor with activity against both *FLT3* internal tandem duplication (ITD) and tyrosine kinase domain (TKD) and weak activity against c-Kit [51]. Gilteritinib was evaluated in the phase 3 ADMIRAL trial, where adults with R/R *FLT3*-mutated AML were randomized in a 2:1 ratio to receive either gilteritinib or salvage chemotherapy. Gilteritinib significantly improved overall survival and remission rates compared to salvage chemotherapy and retained the clinical activity previously exposed to *FLT3* inhibitors [52]. Gilteritinib induces two distinct marrow responses in *FLT3*-mutated AML: responses with and without differentiation. While responses with differentiation happen in around 50% of the cases, clinically significant DS occurs at a lower frequency of 3–5% in patients receiving monotherapy for R/R disease [22].

### 2.9. Quizartinib

Quizartinib is a type II *FLT3* inhibitor that binds adjacent to the ATP-binding domain when the *FLT3* protein is in its inactive conformation and, therefore, lacks activity against TKD-mutant *FLT3*. Quizartinib caused terminal myeloid differentiation of leukemic blasts with a surge in the number of *FLT3*-ITD-retaining neutrophils, and the development of clinical DS [53]. Dermatologic manifestations including sweet’s syndrome (acute febrile neutrophilic dermatosis) have also been reported with *FLT3* inhibitors, including quizartinib [54]. Quizartinib is FDA-approved (since July 2023) in combination with standard induction/consolidation chemotherapy and as maintenance monotherapy for ND *FLT3*-ITD mutated AML in R/R setting. DS incidence is lower (1–5% in real-world and trial data), compared with *IDH* and menin inhibitors and usually presents with dermatologic manifestations (Oncologic Drugs Advisory Committee (ODAC) Meeting, 14 May 2019, NDA 212166, Quizartinib, Daiichi-Sankyo, Inc., Tokyo, Japan)

## 3. Methods

This review was conducted and reported in accordance with the Scale for the Assessment of Narrative Review Articles (SANRA) guidelines [55]. The SANRA criteria guided the formulation of the research questions, the structured literature search strategy, the critical appraisal of included studies, and the organization of evidence into thematic sections relevant to this review. We conducted a structured literature search using the PubMed (MEDLINE) database to identify relevant English-language publications from 2010 to the present (September 2025). This timeframe was chosen to capture the modern era of differentiation-directed treatments in AML, as *IDH*1/2 inhibitors, *FLT3* inhibitors, and menin inhibitors have entered clinical trials and practice mainly in the past decade. The search term strategy employed combinations of keywords related to AML and differentiation therapy, including “acute myeloid leukemia”, “*IDH*1”, “*IDH*2”, “*FLT3*”, “gilteritinib”, “quizartinib”, and “menin inhibitor”. Studies included phase I–III clinical trials and clinical studies evaluating differentiation-directed targeted therapy in AML. A limited number of preclinical or translational studies that were deemed mechanistically essential were also included. Editorials, commentaries, and studies lacking relevance to differentiation mechanisms or clinical outcomes were excluded. Data from the included publications were extracted and synthesized through a qualitative, thematic approach. Evidence was first aggregated to define key biological mechanisms of differentiation blockade in AML and then grouped by therapeutic class. Within each group, we compared study results in terms of clinical efficacy and evidence of differentiation induction. We also extracted reported resistance mechanisms from both clinical studies. Finally, we summarized emerging research on therapeutic strategies to circumvent resistance.

## 4. Conclusions

Differentiation arrest is a central pathogenic feature of AML and a tractable therapeutic target beyond APL. Molecularly targeted agents, including *IDH*1/2 and menin inhibitors and selective *FLT3* inhibitors, restore leukemic maturation through transcriptional and epigenetic reprogramming, producing meaningful clinical responses in the genetically defined AML subset. Differentiation syndrome represents an on-target inflammatory consequence of effective therapy and is manageable with early recognition and corticosteroids. However, incomplete remissions and acquired resistance limit single-agent efficacy. Rational combination strategies, biomarker-driven patient selection, and deeper mechanistic understanding are essential to fully integrate differentiation therapy into precision-based, non-cytotoxic AML treatment paradigms.

## 5. Future Directions

Future directions for differentiation-directed therapy AML increasingly center on the rational integration of menin and *IDH*1/2 inhibitors into frontline combination regimens, with an ongoing pivotal phase III trial (KOMET-017; NCT07007312) evaluating ziftomenib in combination with venetoclax and azacitidine or intensive chemotherapy, and early-phase data (BEAT AML sub-study) supporting the advancement of revumenib-based triplets toward potential registrational studies, with the goal of establishing new standards of care for *NPM1*-mutated and *KMT2A*-rearranged AML. Parallel efforts focus on overcoming resistance through next-generation menin inhibitors and molecular surveillance for emerging MEN1 alterations, with expansion into R/R settings including combination strategies with intensive salvage regimens and *FLT3* inhibitors (KOMET-008; NCT06001788), exploiting transcriptional dependencies such as STAT5A signaling in co-mutated disease. Biomarker development beyond founding driver mutations, particularly HOXA/B and MEIS1 expression signatures, may enable broader patient selection, including NUP98-rearranged leukemias [56]. For *IDH*1/2 inhibitors, future studies emphasize optimized triplet regimens [57], the investigation of epigenetic and metabolic resistance mechanisms, and the evaluation of maintenance strategies to enhance response durability. Collectively, these approaches aim to extend differentiation-based, precision therapies across AML subsets beyond APL.

## Figures and Tables

**Table 1:** List of clinical trials evaluating *IDH*1/2 inhibitors.

Medication	Trial/ID (Ref)	Line of Therapy	Response Rate (ORR/CR/CRh)	Grade ≥ 3 Complications	Differentiation Syndrome (DS)
Ivosidenib	Phase 1 Expansion NCT02093559 (AG120-C-001)	R/R AML	ORR: 41.6% CR/CRh: 30.4% CR: 21.6%	QT Prolongation: 7.8% Leukocytosis: 1.7%	10.6% (Any grade) 3.9% (Grade ≥ 3) FDA review: 19%
Ivosidenib + Azacitidine	Phase 3 AGILE	Newly Diagnosed (Unfit)	ORR: 62.5% CR: 47.2%	Neutropenia: 28% Febrile Neutropenia: 28%	14% (Any grade) No Grade ≥ 4
Ivosidenib + Aza + Venetoclax	Phase 1b/2 Triplet (NCT03471260)	ND AML, R/R AML, or MDS/MPN	ORR: 94% CRc: 93%	Febrile Neutropenia: 28% Infection: 24%	11% (Any grade) No G4/5 reported
Enasidenib	Phase 1/2 Study NCT01915498 (AG221-C-001) (24, 25)	R/R AML	ORR: 40.3% CR: 19.3%	Hyperbilirubinemia: 12% Thrombocytopenia: 6%	12% (Any grade) 7% Grade ≥ 3
Enasidenib + Azacitidine	Phase 1b/2 Study NCT02677922 (Phase 2)	ND AML (Unfit)	ORR: 74% CR: 54%	Neutropenia: 37% Thrombocytopenia: 37%	18% (Any grade) 8% Grade ≥ 3
Olutasidenib	Phase 2 Pivotal 2102-HEM-101 (NCT02719574)	R/R AML	ORR: 48% CR/CRh: 35%	Transaminitis: 13% Febrile Neutropenia: 8%	14% (Any grade) 9% (Grade ≥ 3) 1 Fatal case
Olutasidenib + Azacitidine	NCT02719574 (Pivotal)	R/R AML	ORR: 59% CR/CRh: 27%	Thrombocytopenia: 37% Neutropenia: 24%	9% (Any grade) 5% (Grade 3)

Note: Data derived from clinical trials NCT02093559 (Ivosidenib monotherapy), NCT03173248 (AGILE), NCT03471260 (Triplet), NCT01915498 (Enasidenib monotherapy), NCT02677922 (Enasidenib combo), and NCT02719574 (Olutasidenib). Abbreviations: AML: Acute Myeloid Leukemia; Aza: Azacitidine; CR: Complete Remission; CRh: Complete Remission with partial hematologic recovery; CRc: Composite Complete Remission; DS: Differentiation Syndrome; MDS/MPN: Myelodysplastic Syndrome/Myeloproliferative Neoplasm; ND: Newly Diagnosed; ORR: Overall Response Rate; R/R: Relapsed/Refractory; Grade: Adverse events graded per CTCAE criteria. Differentiation syndrome rates include both investigator-reported and FDA-adjudicated data where specified.

## Data Availability

No new data were created or analyzed in this study.

## Abbreviations

The following abbreviations are used in this manuscript:

7+3	Cytarabine plus anthracycline induction chemotherapy
α-KG	Alpha-ketoglutarate
ADMIRAL	Phase III trial of gilteritinib in relapsed/refractory AML
AGILE	Phase III trial of ivosidenib plus azacitidine
ALL	Acute lymphoblastic leukemia
ALT	Alanine aminotransferase
AML	Acute myeloid leukemia
APL	Acute promyelocytic leukemia
ATRA	All-trans retinoic acid
ATO	Arsenic trioxide
Aza	Azacitidine
BID	Twice daily
CI	Confidence interval
CR	Complete remission
CRc	Composite complete remission
CRh	Complete remission with partial hematologic recovery
CRi	Complete remission with incomplete hematologic recovery
CTCAE	Common Terminology Criteria for Adverse Events
Dec/Ced	Decitabine/cedazuridine
DLT	Dose-limiting toxicity
DS	Differentiation syndrome
EFS	Event-free survival
FDA	U.S. Food and Drug Administration
*FLT3*	FMS-like tyrosine kinase 3
HOX	Homeobox gene family
HR	Hazard ratio
*IDH*1/2	Isocitrate dehydrogenase 1/2
IPSS	International Prognostic Scoring System
ITD	Internal tandem duplication
*KMT2A*	Lysine methyltransferase 2A
*KMT2A*r	KMT2A-rearranged
MEIS1	Myeloid ecotropic viral integration site 1
MEN1	Menin gene
MDS	Myelodysplastic syndrome
MPN	Myeloproliferative neoplasm
MRD	Measurable residual disease
ND	Newly diagnosed
NGS	Next-generation sequencing
*NPM1*	Nucleophosmin 1
*NPM1*m	*NPM1*-mutated
ODAC	Oncologic Drugs Advisory Committee
ORR	Overall response rate
OS	Overall survival
PML–RARA	Promyelocytic leukemia–retinoic acid receptor alpha fusion
QTc	Corrected QT interval
R/R	Relapsed or refractory
RP2D	Recommended phase II dose
SANRA	Scale for the Assessment of Narrative Review Articles
STAT5A	Signal transducer and activator of transcription 5A
TEAE	Treatment-emergent adverse event
TKD	Tyrosine kinase domain
TRAEs	Treatment-related adverse events
Ven	Venetoclax
